# Cateteres venosos centrais de inserção periférica: alternativa ou primeira escolha em acesso vascular?

**DOI:** 10.1590/1677-5449.011516

**Published:** 2017

**Authors:** Marcelo Kalil Di Santo, Diogo Takemoto, Robert Guimarães Nascimento, Ariele Milano Nascimento, Érika Siqueira, Caio Túlio Duarte, Marco Antônio Caldas Jovino, Jorge Agle Kalil

**Affiliations:** 1 Rede D’or Hospital e Maternidade São Luiz – HMSL Itaim, Serviço de Cirurgia Vascular e Endovascular, São Paulo, SP, Brasil.; 2 Hospital da Beneficência Portuguesa de São Paulo, Cirurgia Geral, São Paulo, SP, Brasil.; 3 Rede D’or Hospital e Maternidade São Luiz – HMSL Itaim, Terapia Infusional, São Paulo, SP, Brasil.; 4 Centro Universitário São Camilo – CUSC, São Paulo, SP, Brasil.

**Keywords:** cateter central, acesso venoso central, punção ecoguiada, acesso vascular

## Abstract

**Contexto:**

Os cateteres venosos centrais de inserção periférica (PICC) são dispositivos intravenosos, introduzidos através de uma veia superficial ou profunda da extremidade superior ou inferior até o terço distal da veia cava superior ou proximal da veia cava inferior. Apresentam maior segurança para infusão de soluções vesicantes/irritantes e hiperosmolares, antibioticoterapia, nutrição parenteral prolongada (NPT) e uso de quimioterápicos; demonstram reduzido risco de infecção em comparação a outros cateteres vasculares e maior relação custo/benefício se comparados ao cateter venoso de inserção central (CVCIC).

**Objetivos:**

Apresentar os resultados de implantes de PICCs ecoguiados e posicionados por fluoroscopia realizados no Hospital e Maternidade São Luiz (HMSL) Itaim, Rede D’or, Brasil.

**Métodos:**

Estudo prospectivo, não randomizado, realizado entre fevereiro de 2015 e novembro de 2016. Utilizou-se protocolo pré-estabelecido pela instituição em casos de solicitação de acesso vascular. Foram analisadas indicações, doenças prevalentes, tipo do cateter implantado, sucesso técnico, complicações relacionadas ao cateter, e estabelecidos critérios de inclusão e exclusão.

**Resultados:**

Solicitados 256 acessos vasculares, sendo implantados 236 PICCs (92,1%) e 20 CVCICs (7,9%). Principais indicações: antibioticoterapia prolongada (52,0%), NPT (19,3%) e acesso venoso difícil (16,0%). Houve sucesso técnico em 246 cateteres implantados (96,1%). A veia basílica direita foi a principal veia puncionada em 192 pacientes (75,0%), seguida da braquial direita em 28 pacientes (10,9%).

**Conclusões:**

O implante dos PICCs ecoguiados e posicionados por fluoroscopia demonstrou baixa incidência de complicações, reduzidos índices de infecção e é seguro e eficaz em casos de acessos vasculares difíceis, sendo esses cateteres considerados dispositivos de escolha em acesso vascular central.

## INTRODUÇÃO

O cateter venoso central de inserção periférica (*peripherally inserted central catheter*, PICC) é um dispositivo intravenoso inserido através de uma veia superficial ou profunda da extremidade e que progride até o terço distal da veia cava superior ou proximal da veia cava inferior. Pode medir de 20 a 65 cm de comprimento, com calibre variando de 1 a 6 French (Fr). Possui de um a três lumens e pode ser valvulado (proximal ou distal) ou não valvulado. É flexível, radiopaco, de paredes lisas e homogêneas, e confeccionado em silicone, polietileno, poliuretano, ou carbotano. É inserido por punção percutânea através de agulhas bipartidas, metálicas ou plásticas, para descarte posterior.

O PICC foi descrito na literatura pela primeira vez em 1929 pelo médico alemão Werner Theodor Otto Forssmann ao inserir uma cânula em sua própria veia antecubital, através da qual introduziu um cateter de 65 cm até o átrio direito, sendo confirmada a localização anatômica por imagem radiográfica. O procedimento rendeu-lhe o prêmio Nobel de Medicina em 1956, surgindo então uma alternativa de acesso venoso central por via periférica^1^. No Brasil, começou a ser utilizado na década de 1990, a princípio para uso em neonatologia, devido ao pequeno diâmetro do cateter e à flexibilidade do material (silicone), sendo posteriormente empregado em larga escala em terapia intensiva, oncologia e cuidados domiciliares^2^.

O dispositivo apresenta indicações e contraindicações estabelecidas; preconizamos implante guiado por ultrassonografia e posicionamento guiado por fluoroscopia, assegurando, dessa forma, maior segurança durante a punção e o posicionamento do implante, oferecendo maior conforto para o paciente durante o procedimento.

As principais vantagens dos PICCs são: o benefício de inserção do cateter sob anestesia local, associada ou não à sedação; redução do desconforto do paciente, evitando múltiplas punções venosas; possibilidade de ser inserido à beira do leito; obter via segura para administração de antibióticos; nutrição parenteral prolongada (NPT); excelente via para quimioterápicos; maior tempo de permanência e menor risco de contaminação em relação a outros dispositivos; preservação do sistema venoso periférico; e possível indicação de terapia domiciliar.

Um aspecto de fundamental importância na prevenção de complicações e iatrogenias é o fato de o cateter ter inserção periférica, o que potencialmente evita a ocorrência de pneumotórax e hemotórax. Além disso, tem custo inferior ao do cateter venoso central inserido cirurgicamente (CVCIC)^3^^,^ 4.

As principais dificuldades e desvantagens do uso dos PICCs estão relacionadas à necessidade de uma rede vascular íntegra e calibrosa para o implante; necessidade de treinamento especial para inserção e manutenção do cateter; monitorização rigorosa do dispositivo; e necessidade de radiografia para localização da ponta do cateter^3^^,^ 4. Evidências demonstraram que o dispositivo não é isento de complicações, tais como trombose venosa profunda (TVP), tromboflebites, oclusões do cateter, pseudoaneurismas arteriais e infecções^5^^-^ 8. Por outro lado, o emprego desse cateter evita a dissecção venosa e apresenta menor exposição do paciente a dor e complicações inerentes ao procedimento.

O objetivo deste estudo foi apresentar os resultados de nosso grupo no implante de PICCs ecoguiados e posicionados por fluoroscopia realizados no Hospital e Maternidade São Luiz (HMSL) Itaim, Rede D’or, São Paulo, SP, Brasil.

## MÉTODOS

**Figura 1:**
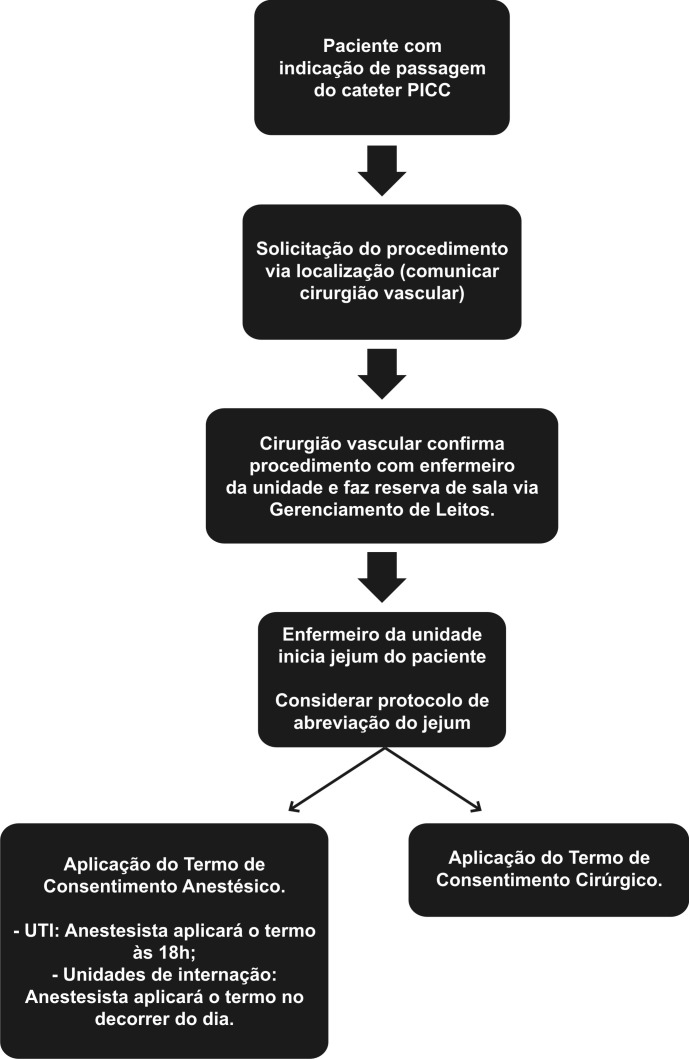
Fluxograma estabelecido para solicitação de passagem de cateter PICC.

Elaboramos um estudo prospectivo, não randomizado, realizado de fevereiro de 2015 a novembro de 2016, aprovado pelo Comitê de Ética em Pesquisa. Foram utilizados protocolos pré-estabelecidos pela instituição, em pacientes com solicitação e indicação de acesso vascular (Figuras 1 e 2). Os critérios de inclusão estabelecidos foram: pacientes internados em enfermarias ou unidade de terapia intensiva (UTI) com indicação de NPT, infusão de drogas vesicantes e/ou irritantes, acesso difícil com perda de acesso diária, quimioterapia, antibioticoterapia prolongada por período acima de 4 dias e pacientes em uso de heparina e/ou plaquetopênicos. Contraindicação para a passagem do cateter e/ou para o estudo incluíram pacientes pediátricos, tromboflebite ou TVP de membro superior bilateral, veia cefálica como única opção de acesso bilateralmente, mulheres mastectomizadas, presença de fístulas arteriovenosas no membro a ser puncionado/cateterizado e situações de emergência. O presente estudo analisou: indicações, doenças prevalentes, tipo do cateter implantado, sucesso técnico e complicações relacionadas ao cateter.

**Figura 2:**
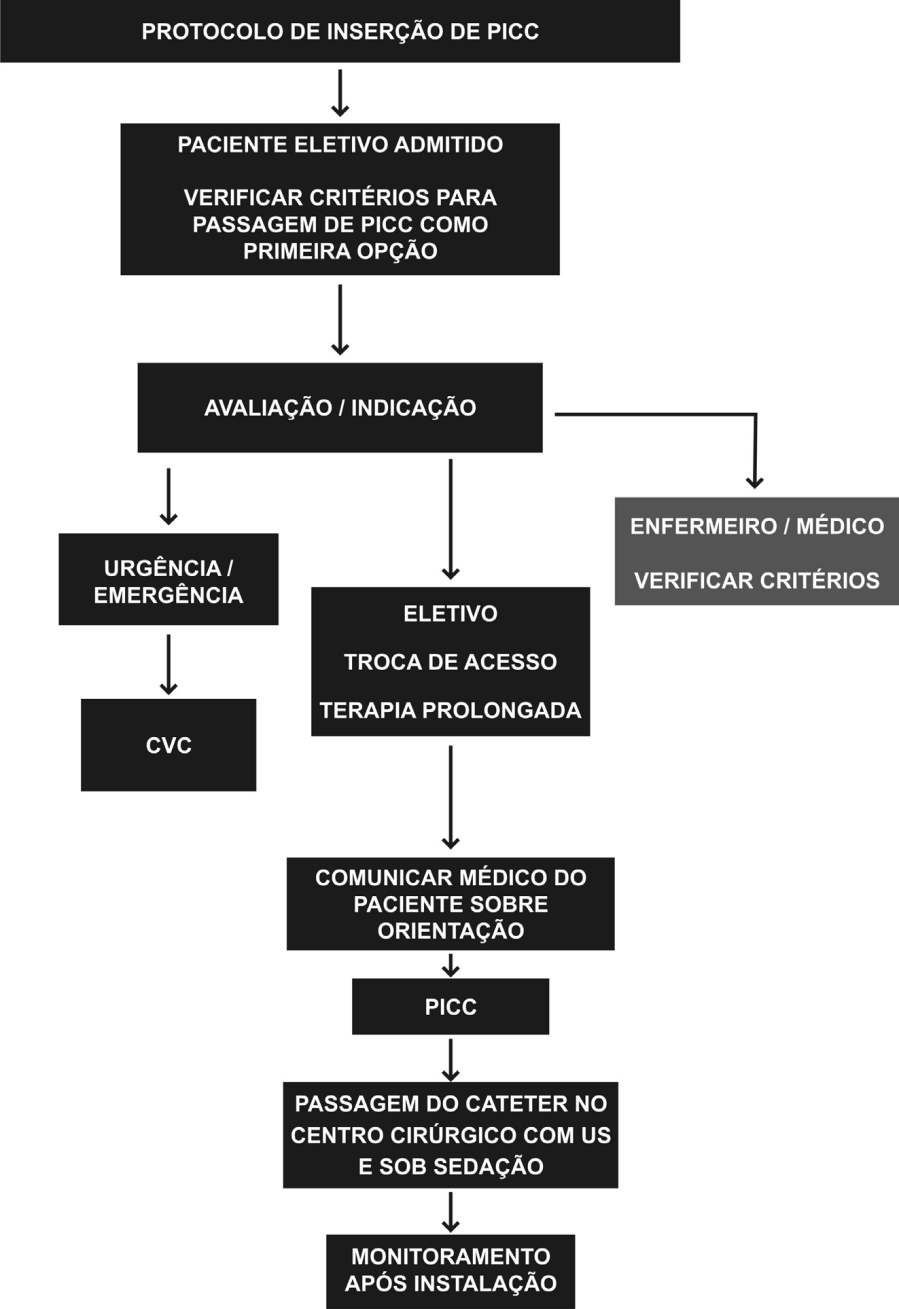
Protocolo de inserção do cateter PICC após solicitação de passagem do cateter. CVC = cateter venoso central; US = ultrassonografia.

A metodologia de punção utilizada em nosso estudo baseia-se na técnica de Seldinger modificada, ecoguiada. O procedimento foi realizado em centro cirúrgico, com o paciente mantido em decúbito dorsal horizontal sob anestesia local ou sob anestesia local e sedação. Foram realizadas assepsia e antissepsia prévias do braço selecionado com digliconato de clorexidina 2,0% (solução com tensoativos, conjunto escova-esponja) e o paciente foi completamente coberto com campos estéreis.

A veia periférica adequada do membro superior foi selecionada e puncionada utilizando o aparelho de ultrassom em modo B (Mindray – Hemocat®).

**Figura 3:**
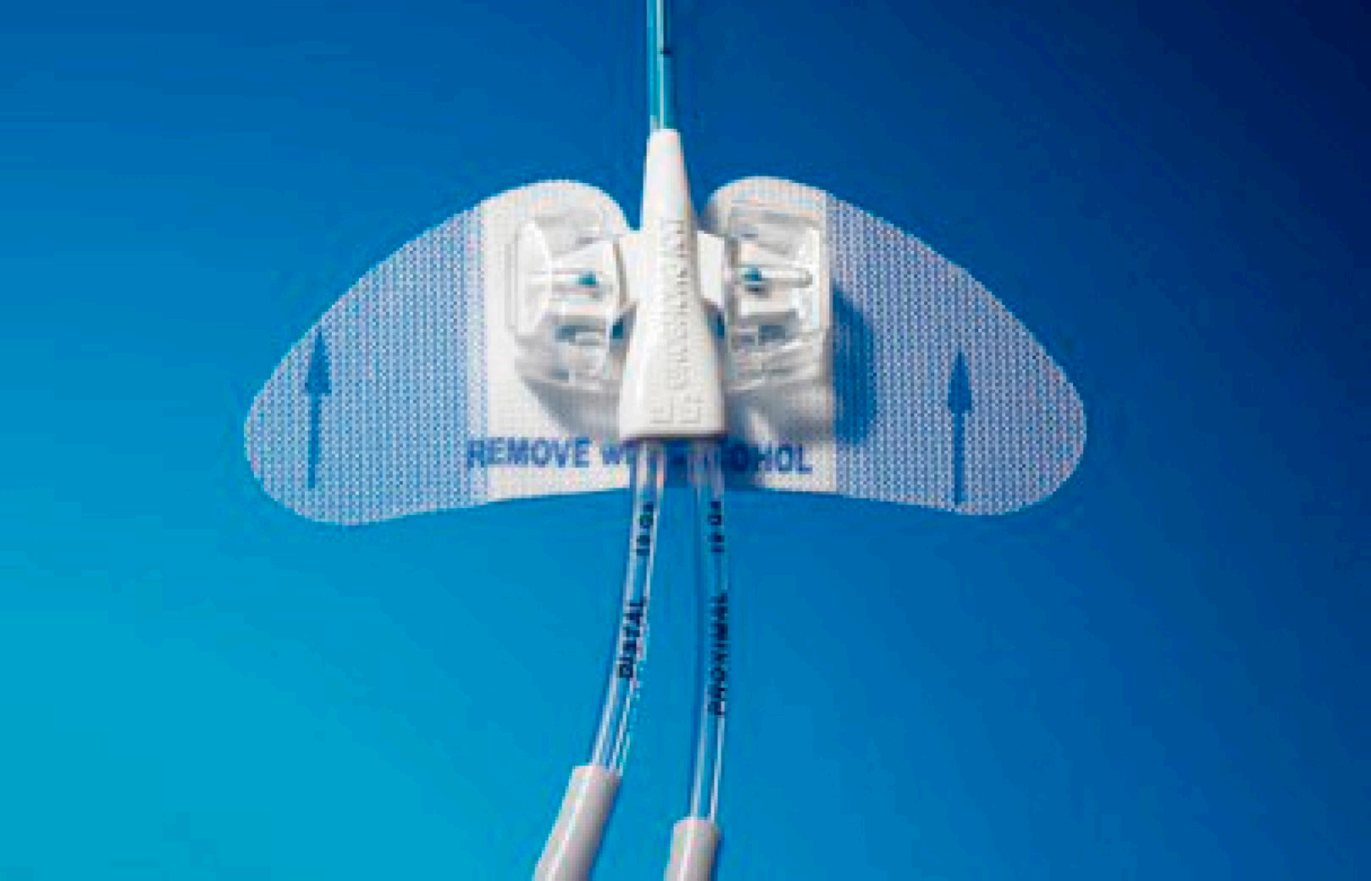
Dispositivo de fixação do cateter.

Optamos pelo tipo de punção fora do plano (*out-of-plane puncture*); a escolha do sítio de punção adequado no membro superior foi realizada conforme proposto por Dawson^9^, delimitando as zonas ideais de inserção sob orientação ultrassonográfica (Zone Insertion Method, ZIM). Após a locação do fio guia metálico centimetrado, inseriu-se a bainha dilatadora (Peel*-*Away®); o cateter selecionado foi inserido após secção em comprimento, com as respectivas medidas prévias adequadas. A extensão final do cateter foi baseada no comprimento do fio guia centimetrado. A próxima etapa do procedimento foi avaliar fluxo e refluxo pelo cateter; a seguir, realizar angiografia transoperatória de posicionamento com a finalidade de verificar a localização da ponta; e, finalmente, fixar o cateter com o dispositivo Statlock® (Figuras 3 e 4).

**Figura 4:**
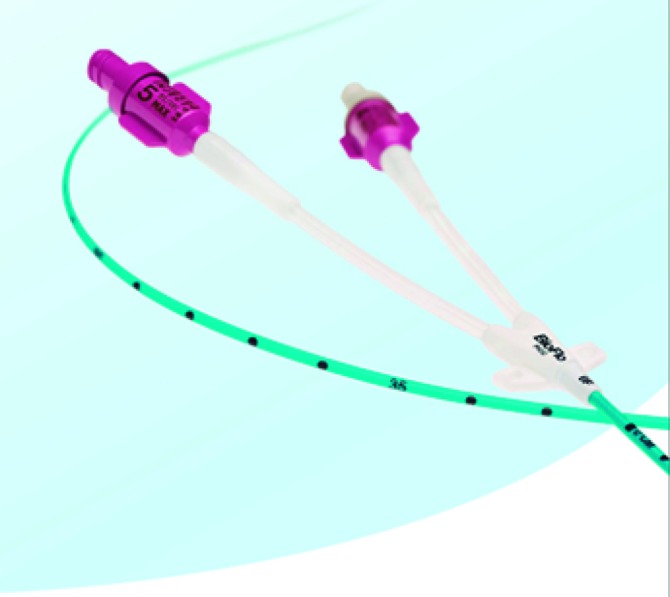
Cateter venoso central de inserção periférica confeccionado em carbotano com sistema de válvula proximal ativado por pressão (PASV) e tecnologia antitrombótica com polímero Endexo®.

## RESULTADOS

Foram solicitados 256 acessos vasculares, sendo implantados 236 PICCs (92,1%) e 20 CVCICs (7,9%). Eram 155 pacientes do sexo feminino (60,5%) e 101 do sexo masculino (39,5%), com média de idade de 70,2 anos. Em relação à procedência no hospital, 176 pacientes estavam internados na UTI (68,7%) e 80 na enfermaria (31,3%). As principais indicações para o implante do cateter foram: antibioticoterapia prolongada (52,0%), NPT (19,3%) e acesso venoso difícil (16,0%). Outras indicações em menor escala foram administração de medicações vesicantes/irritantes (8,0%), risco de sangramento (3,3%) e administração de quimioterápicos (1,4%).

Os cateteres utilizados foram valvulados de silicone (PICC Groshong BARD®), não valvulados de poliuretano (Power PICC BARD®) e valvulados de carbotano (Bioflo Hemocat®) de 5 a 6 Fr.

As doenças clínicas mais encontradas nos pacientes submetidos ao acesso vascular, em ordem de prevalência, estão descritas na Tabela 1.

**Tabela 1:** Doenças mais frequentes por ordem de prevalência.

**Doenças prevalentes**	**Prevalência (total = 256)**
Broncopneumonia	40 (15,6%)
ITU sem pielonefrite	24 (9,4%)
Erisipela membros inferiores	16 (7,5%)
Insuficiência renal crônica	16 (7,5%)
Hiperemese gravídica	16 (7,5%)

ITU: infecção do trato urinário.

Obteve-se sucesso técnico em 246 cateteres implantados (96,1%), definido como posicionamento do cateter no interior da veia cava superior.

Em 10 cateteres (3,9%), não foi possível o posicionamento adequado no interior dessa veia devido a falhas técnicas durante a curva de aprendizado inicial: comprimento inadequado do cateter (doentes com indicação de NPT que obrigatoriamente devem ter PICCs posicionados na veia cava superior ou inferior) e não progressão do cateter apesar de perviedade venosa adequada (como por exemplo dificuldades pelo atrito valvular).

Em 192 pacientes (75,0%) a veia eleita para a inserção foi a veia basílica direita, seguida da veia braquial direita em 28 pacientes (10,9%), veia braquial esquerda em 19 pacientes (7,4%) e, como última opção, veia basílica esquerda em 17 (6,7%).

Ocorreram em nossa casuística 14 complicações relacionadas ao procedimento, dentre as quais duas fraturas com cateteres valvulados distais (0,8%), sete obstruções de cateter (2,7%), sendo seis com cateteres de poliuretano não valvulados e uma com cateter valvulado de carbotano, e cinco infecções, todas estas relacionadas aos cateteres não valvulados (1,9%).

Foram isolados três micro-organismos diferentes: a *Klebsiella pneumoniae* em três casos, *Candida Glabrata* em um caso, e *Staphylococcus hominis* em um caso. Todos os cateteres infectados estavam implantados em doentes da UTI.

## DISCUSSÃO

**Figura 5:**
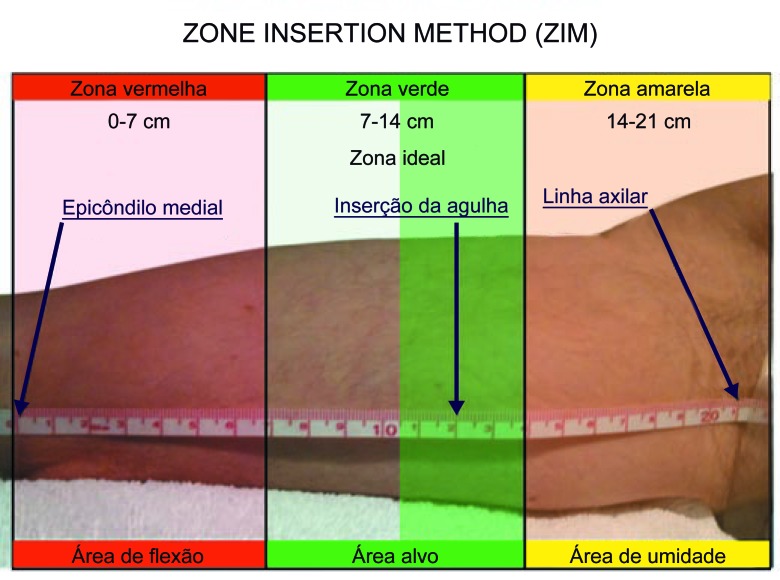
Exemplo de medida de zona total de aproximadamente 21 cm dividida em três zonas de 7 cm cada nas respectivas cores vermelha, verde e amarela.

Robert B. Dawson delimitou zonas ideais de inserção do PICC sob orientação ultrassonográfica (ZIM). Através de características musculoesqueléticas da pele e dos vasos, delimitou o braço acima da prega antecubital em três zonas distintas, de 7 cm cada, separadas pelas cores vermelho, verde e amarelo, tendo como referência anatômica inicial o epicôndilo medial do úmero e como final a linha axilar (Figura 5). Tal como o sistema de semáforos, a cor da zona indica se uma zona deve ou não ser utilizada para punção. Segundo o autor, a zona ideal a ser puncionada é referida pela cor verde, encontrando-se a aproximadamente 12 cm do epicôndilo medial, região onde a veia basílica estaria mais superficial em relação ao plano da pele^9^.

Em nosso estudo, o implante do PICC ecoguiado obteve uma alta taxa de sucesso técnico (96,1%). Nossa preferência pela punção ecoguiada como via de acesso venoso baseou-se no menor risco de punção inadequada, proporcionado pela ultrassonografia, quando comparada com a punção baseada somente em parâmetros anatômicos^10^^,^ 11. Segundo Hockley et al., a literatura mostra que o implante ecoguiado no braço melhora a taxa de sucesso na inserção do cateter^12^^,^ 13 e a satisfação do paciente submetido ao procedimento^14^ e reduz as complicações, como infecção no sítio de punção, trombose e migração do cateter^15^.

Os PICCs podem ter como principais complicações: infecção, fratura com migração venosa para distal, tromboflebite ou TVP em extremidade superior, síndrome de Horner e até mesmo quilotórax^16^^-^ 18, dentre as quais as mais comumente encontradas são infecções, tromboflebites e TVP^5^^-^ 8.

**Figura 6:**
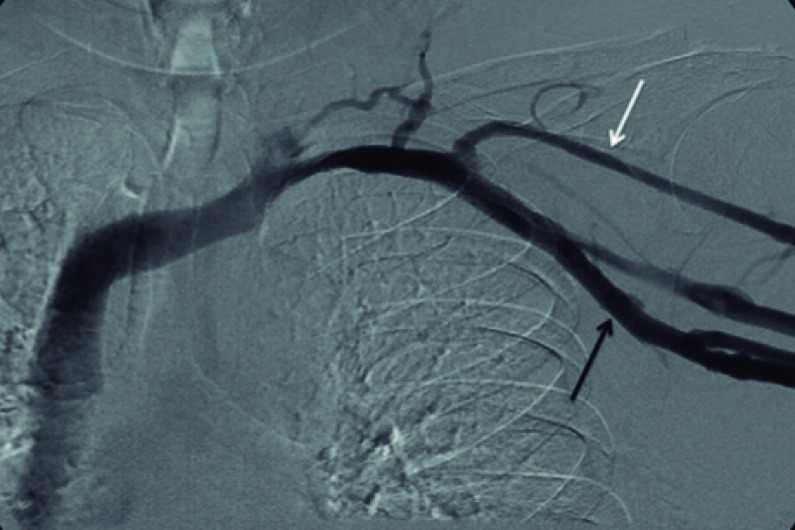
Menor diâmetro da veia cefálica (seta branca) em relação ao tamanho do cateter e sua inserção perpendicular na veia axilar (seta preta).

Segundo estudo de Liem et al.^19^, as taxas de trombose venosa superficial sintomática em extremidade superior associadas aos PICCs corresponderam a 1,9% para veia basílica, 7,2% para veia cefálica, e 0% para veia braquial. A maior incidência de trombose venosa superficial na veia cefálica deve-se às características anatômicas próprias do vaso, como o menor diâmetro em relação ao tamanho do cateter, menor número de tributárias e uma inserção mais perpendicular na veia axilar (Figura 6).

Por esse motivo, nosso grupo adotou como critério de exclusão a veia cefálica como única opção de acesso bilateral, optando, portanto, pelo implante do CVCIC nesses casos.

A revisão de estudos retrospectivos e prospectivos publicados demonstrou incidência de TVP de membros superiores relacionado ao PICC que variou de 0,5 a 19,4%, sendo que as incidências mais altas foram diretamente relacionadas ao implante de PICCs de diâmetros maiores e a presença de neoplasias malignas. Apenas um único paciente oncológico da nossa casuística, também portador de TVP de membros inferiores, apresentou quadro de TVP de membro superior evidenciado em cateter não valvulado, que foi tratado com a remoção do cateter, não sendo necessária a anticoagulação subcutânea ou oral.

PICCs com tecnologias de válvulas integradas reduziram significativamente as taxas de complicações tardias (oclusão ou infecção) em comparação com PICCs não valvulados, assim como eliminaram a necessidade do uso de heparina e suas potenciais complicações subsequentes relacionadas (por exemplo, trombocitopenia induzida por heparina)^20^.

Estudo retrospectivo realizado pela Universidade Vanderbilt que envolveu 12.505 dispositivos implantados, comparando os índices de infecção e oclusão em PICCs valvulados (4,2% e 1,4% respectivamente) e não valvulados (5,5% e 6,3% respectivamente), concluiu que PICCs valvulados apresentaram menores taxas de infecção e oclusão, menor necessidade de manutenção e por fim, menor custo, substituindo os preenchimentos de heparina obrigatórios, exigidos por alguns PICCs não valvulados^21^. Os resultados obtidos em nosso estudo referentes às sete obstruções descritas (seis delas em cateteres não valvulados e apenas uma em cateter valvulado) estão de acordo com tais dados publicados na literatura^20^^,^ 21.

PICCs com válvulas proximais e distais foram introduzidos no mercado na tentativa de reduzir oclusões de cateter por meio da prevenção do fluxo sanguíneo retrógrado^22^. Estudo prospectivo randomizado conduzido por Hoffer et al.^22^ apontou maior taxa de perviedade dos cateteres valvulados proximais, com menor incidência de complicações oclusivas e infecciosas em comparação aos cateteres valvulados distais.

A complicação mais comum durante a inserção é o mal posicionamento de cateteres, ocorrendo quando o cateter não atinge o local apropriado dentro da veia cava^23^. Dificuldade na progressão do cateter durante a inserção, aspiração sanguínea inadequada e dificuldades na remoção do estilete/bainha dilatadora são indicações de que pode ter ocorrido mal implante do cateter e, nesses casos, o emprego da radiografia ou fluoroscopia é imprescindível para identificar se o cateter foi mal posionado^24^.

**Figura 7:**
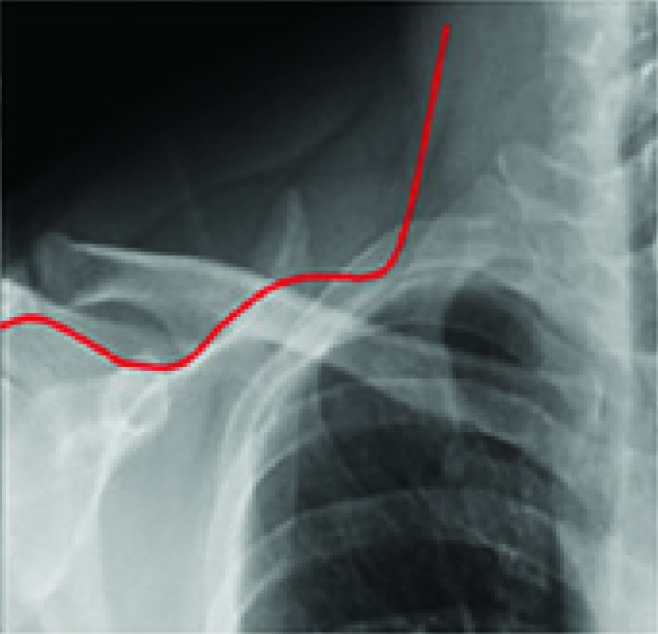
Trajeto de cateter posicionado inadequadamente em veia jugular interna.

Em estudo reunindo 3.012 pacientes, realizado por Song e Li^23^, obteve-se sucesso técnico de 94,6% na implantação dos PICCs e foram identificados 237 dispositivos posicionados inadequadamente fora da veia cava, avaliados por radiografia após inserção do cateter, sendo o local mais frequente a veia jugular, seguida da veia axilar e braquial (Figura 7). Nosso serviço, mediante protocolo pré-estabelecido, não demonstrou cateteres mal posicionados. Ao identificarmos local diferente da posição central pelo uso da fluoroscopia, os cateteres eram imediatamente reposicionados.

Na ocorrência de obstruções de cateter, é necessário instituir a terapia medicamentosa adequada com o uso de trombolíticos, visando reduzir o grau de obstrução do cateter. Estudo realizado por Baskin et al.^25^ demonstra que agentes trombolíticos eliminam com êxito oclusões de cateter na maioria dos casos, destacando o papel da alteplase. Descrita como medicamento seguro e efetivo na desobstrução de cateteres, a droga apresenta como desvantagens o maior custo, ser menos efetiva que a uroquinase nos primeiros 30 minutos de infusão e demandar tempo superior a 4 horas para alcançar a depuração do cateter. Outros agentes trombolíticos requerem menor tempo de permanência no lúmen do vaso para ação adequada^25^. Em nossa casuística, as obstruções evidenciadas com os seis cateteres não valvulados e um valvulado proximal foram adequadamente tratadas utilizando a uroquinase (Taurolock®), trombolítico de escolha em nossa instituição.

Ao examinar o risco de infecção de corrente sanguínea em diferentes tipos de dispositivo, Maki et al.^26^ encontraram taxas de infecção de corrente sanguínea associadas a PICC inferiores às relatadas com dispositivos venosos centrais tradicionais, não tunelizados.

Muitas hipóteses, incluindo menor densidade bacteriana na pele sobre o braço, temperaturas mais frias nas extremidades e relativa facilidade de cuidado do local em comparação ao pescoço e à virilha, foram sugeridas para explicar o fato de que os PICCs exibiram menores complicações infecciosas em relação aos outros dispositivos^6^. Estão relacionados à infecção de corrente sanguínea associada ao PICC: tempo de internação hospitalar, internação em UTI e número de lumens do dispositivo^27^. Em estudo realizado Sundriyal et al.^28^, em que foram implantados 246 PICCs em UTIs, observou-se que 12,5% apresentaram infecção de cateter com hemocultura positiva, sendo os agentes mais comuns isolados *Klebsiella pneumoniae* e *Staphylococcus sp.* Os autores sugerem que todos os cateteres infectados sejam retirados em pacientes com hemoculturas positivas, principalmente se não houve melhora do quadro febril após 48 h de administração adequada de antibióticos^28^. Fungemia ou bacteremias com espécies de *Bacilus, Corynebacterium jeikeium, Staphylococcus aureus, Pseudomonas aeruginosa* ou *Stenotrophomonas maltophila* e micobactérias não tuberculosas muitas vezes persistem, apesar dos antibióticos apropriados, e requerem remoção do cateter. A remoção do cateter também deve ser considerada quando hemoculturas permanecem positivas após 48 h de tratamento com antibióticos; se não houver outro sítio de infecção identificado, ou se a bacteremia repetir-se após a conclusão de um curso de antibióticos^29^.

Em nossa casuística houve cinco casos de infecção de cateter com posterior retirada, devido aos principais agentes encontrados: *Klebsiella pneumoniae, Candida glabrata* e *Staphylococcus hominis*. Os resultados encontrados em nosso estudo estão de acordo com a literatura.

## CONCLUSÃO

O implante de cateteres venosos centrais de inserção periférica ecoguiados e posicionados por fluoroscopia apresenta baixa incidência de complicações, reduzidos índices de infecção, é seguro e eficaz, principalmente nos casos de acessos vasculares difíceis, sendo esses cateteres considerados os dispositivos de escolha em acesso vascular central. Sua manutenção necessita de treinamento rigoroso da equipe de enfermagem, que deve ser responsável apenas por preservar, zelar e salvaguardar o cateter, com a finalidade de minimizar complicações pela manipulação inadequada deste. O procedimento deve ser realizado por médico treinado, capaz de conduzir e resolver eventuais complicações relacionadas à inserção e utilização do cateter.
