# Supplementary material for: Cateteres venosos centrais de inserção periférica: alternativa ou primeira escolha em acesso vascular?
Source: J Vasc Bras. 2017 Apr-Jun;16(2):104–12. [Article in Portuguese] doi: 10.1590/1677-5449.011516 (PMC5915858; doi:10.1590/1677-5449.011516)
Supplement: Translation

# Translation: Peripherally inserted central venous catheters: alternative or first choice vascular access?

**Authors:** Marcelo Kalil Di Santo, Diogo Takemoto, Robert Guimarães Nascimento, Ariele Milano Nascimento, Érika Siqueira, Caio Túlio Duarte, Marco Antônio Caldas Jovino, Jorge Agle Kalil

**Affiliations:** 1 Rede D’or Hospital e Maternidade São Luiz – HMSL Itaim, Serviço de Cirurgia Vascular e Endovascular, São Paulo, SP, Brazil.; 2 Hospital da Beneficência Portuguesa de São Paulo, Cirurgia Geral, São Paulo, SP, Brazil.; 3 Rede D’or Hospital e Maternidade São Luiz – HMSL Itaim, Terapia Infusional, São Paulo, SP, Brazil.; 4 Centro Universitário São Camilo – CUSC, São Paulo, SP, Brazil.

**Keywords:** central catheter, central venous access, ultrasound-guided puncture, vascular access

## Abstract

**Background:**

Peripherally inserted central catheters (PICC) are intravenous devices inserted through a superficial or deep vein of an upper or lower extremity and advanced to the distal third of the superior vena cava or proximal third of the inferior vena cava. They offer the advantages of greater safety for infusion of vesicant/irritant and hyperosmolar solutions and enable administration of antibiotics, prolonged parenteral nutrition (PPN), and chemotherapy agents. They also involve reduced risk of infection compared to other vascular catheters and are more cost-effective than centrally inserted venous catheters (CICVC).

**Objectives:**

To present the results of our team’s experience with US-guided and fluoroscopy-positioned PICC placement at the Hospital and Maternidade São Luiz (HMSL) Itaim, Rede D’or, Brazil.

**Methods:**

This was a prospective, non-randomized study, conducted from February 2015 to November 2016. The institution’s preestablished protocol was followed when vascular access was requested. Indications, prevalent diseases, type of catheter implanted, technical success, and complications related to the catheters were analyzed and inclusion and exclusion criteria are described.

**Results:**

A total of 256 vascular accesses were requested, and 236 PICCs (92.1%) and 20 CICVCs (7.9%) were implanted. The main indications were as follows: prolonged antibiotic therapy (52%), PPN (19.3%), and difficult venous access (16%). Technical successes was achieved in 246 catheter placements (96.1%). The right basilic vein was the most common vein punctured for access, in 192 patients (75%), followed by the right brachial vein, in 28 patients (10.9%).

**Conclusions:**

Ultrasound-guided and fluoroscopy-positioned PICC placement had a low incidence of complications, reduced infection rates, and proved safe and effective in cases of difficult vascular access. PICCs can be considered the devices of choice for central vascular access.

## INTRODUCTION

Peripherally inserted central catheters (PICC) are intravenous devices that are inserted via a superficial or deep vein in an extremity and advanced as far as the distal third of the superior vena cava or proximal third of the inferior vena cava. They can measure from 20 to 65 cm in length and have calibers varying from 1 to 6 French (Fr). They can have from one to three lumens and may be valved (proximal or distal) or nonvalved. They are flexible and radiopaque, have smooth, uniform walls, and can be made from silicone, polyethylene, polyurethane, or carbothane. They are inserted by percutaneous puncture using split sheaths, made from metal or plastic, and are discarded after use.

A PICC was described in the literature for the first time in 1929 by the German doctor Werner Theodor Otto Forssmann who inserted a cannula into his own antecubital vein and used it to introduce a 65 cm catheter up to the right atrium, confirming the anatomic location by X-ray. This procedure earned him the 1956 Nobel prize for medicine and introduced an alternative option for central venous access via a peripheral access.^1^ The technique began to be used in Brazil in the 1990s, initially for applications in neonatology, because of the small diameter of the catheter and the flexibility of the material (silicone), and was later widely adopted in intensive care, oncology, and home care.^2^

Indications and contraindications for the device have been established; it is recommended that insertion be guided using ultrasonography and positioning of the tip guided with fluoroscopy, thereby ensuring greater safety during puncture and positioning and increasing patient comfort during the procedure.

The principal advantages of PICCs are as follows: the benefits of inserting the catheter under local anesthesia, combined or not with sedation; reduction of patient discomfort, by avoiding multiple vein punctures; the possibility of bedside insertion; provision of a safe access for administration of antibiotics; prolonged parenteral nutrition (PPN); an excellent access for administering chemotherapy; increased maximum indwell time and reduced risk of contamination compared with other devices; preservation of the peripheral venous system; and possibility of use in home treatment applications.

One feature of fundamental importance to prevention of complications and iatrogenic events is the fact that the catheter is inserted peripherally, which can potentially prevent occurrence of pneumothorax or hemothorax. Additionally, they are less expensive than surgically inserted central venous catheters (SICVCs).^3^^,^ 4

The main difficulties and disadvantages with using PICCs are related to the need for an intact vascular network with sufficient caliber for implantation; the need for special training for insertion and maintenance of the catheter; the need for rigorous monitoring of the device; and the need for radiography to locate the tip of the catheter.^3^^,^ 4 Evidence has shown that these devices are not free from complications, such as deep venous thrombosis (DVT), thrombophlebitis, occlusion of the catheter, arterial pseudoaneurysms, and infections.^5^^-^ 8 On the other hand, using this type of catheter avoids venous dissection and exposes the patient to less pain and fewer complications inherent to the procedure.

To present the results of our team’s experience with US-guided and fluoroscopy-positioned PICC placement at the Hospital and Maternidade São Luiz (HMSL) Itaim, Rede D’or, São Paulo, SP, Brazil.

## METHODS

We conducted a prospective, non-randomized study from February 2015 to November 2016, with Research Ethics Committee approval. Our institution’s preestablished protocol was followed whenever vascular access was requested (Figures 1 and 2). The inclusion criteria adopted were: patients admitted to wards or the intensive care unit (ICU) with indications for PPN, infusion of vesicant and/or irritant drugs, difficult access with loss of daily access, chemotherapy, prolonged antibiotic therapy for periods greater than 4 days, and patients on heparin and/or with thrombocytopenia. Contraindications for catheter insertion and/or for the study included pediatric patients, bilateral thrombophlebitis or DVT of upper extremities, cephalic vein as only access option bilaterally, women with mastectomies, presence of arteriovenous fistulas in the extremity to be punctured/catheterized and emergency situations. The following variables were analyzed: indications, prevalent diseases, type of catheter implanted, technical success, and complications related to the catheter.

**Figure 1:** Flow diagram illustrating procedure for requesting placement of PICC line.

**Figure 2:** Protocol for PICC line insertion after request for catheter placement. CVC = central venous catheter; US = ultrasonography.

The puncture methodology employed in this study is based on the Seldinger technique, modified for US-guidance. The procedure was conducted in an operating theater, with the patient in horizontal decubitus dorsal under local anesthesia or local anesthesia and sedation. Asepsis and antisepsis of the arm chosen were conducted in advance using 2.0% chlorhexidine digluconate (solution containing tensoactive agents, sponge and brush) and the patient was completely covered with sterile drapes and fields.

An appropriate peripheral vein in the upper extremity was selected and punctured with the aid of an ultrasound unit in B mode (Mindray – Hemocat®).

We used out-of-plane puncture; the appropriate puncture site on the upper limb was chosen as proposed by Dawson,^9^ delimiting ideal zones for insertion with ultrasound guidance (the Zone Insertion Method, ZIM). After placement of a metallic guidewire graduated in centimeters, the (Peel*-*Away®) dilation sheath was inserted and then the selected catheter was inserted after sectioning to length, with the appropriate preparatory measures. The final length of the catheter was calculated using the length of the graduated guidewire. The next stage of the procedure is to evaluate flow and backflow through the catheter; followed by transoperative angiography to test the positioning and check that the tip is correctly placed before fixing the catheter with a Statlock® device (Figures 3 and 4).

**Figure 3:** Catheter fixation device.

**Figure 4:** Peripheral central venous catheter made from carbothane with proximal pressure-activated safety valve (PASV) and Endexo® polymer antithrombotic technology.

## RESULTS

During the study, 256 vascular accesses were requested and 236 PICCs (92.1%) and 20 SICVCs (7.9%) were implanted. There were 155 female patients (60.5%) and 101 male patients (39.5%), with a mean age of 70.2 years. Within the hospital, 176 patients were in the ICU (68.7%) and 80 were in wards (31.3%). The most common indications for catheter placement were: prolonged antibiotic therapy (52.0%), PPN (19.3%), and difficult venous access (16.0%). Other indications seen with lower frequency were administration of vesicant/irritant medications (8.0%), risk of bleeding (3.3%), and administration of chemotherapy (1.4%).

The catheters used were silicone with valves (PICC Groshong BARD®), polyurethane without valves (Power PICC BARD®), or carbothane with valves (Bioflo Hemocat®), from 5 to 6 Fr.

The clinical diseases most often seen in the patients who underwent vascular access are shown in Table 1 in order of prevalence.

**Table 1:** Most frequent diseases in order of prevalence.

**Prevalent diseases**	**Prevalence (total = 256)**
Bronchopneumonia	40 (15.6%)
UTI without pyelonephritis	24 (9.4%)
Lower limb erysipelas	16 (7.5%)
Chronic renal failure	16 (7.5%)
Hyperemesis gravidarum	16 (7.5%)

UTI: urinary tract infection.

The placement procedure for 246 catheters (96.1%) was technically successful, defined as achieving a catheter position in the interior of the superior vena cava.

In 10 catheters (3.9%) it was not possible to achieve an adequate position within the interior of this vein due to technical failures during the initial learning curve: incorrect catheter length (PPN patients in whom a PICC position in the superior or inferior vena cava is obligatory) and failure to advance the catheter despite adequate vein patency (such as, for example, difficulties caused by valve friction).

In 192 patients (75.0%), the right basilic vein was chosen for insertion, followed, in descending order of frequency, by the right brachial vein in 28 patients (10.9%), the left brachial vein in 19 patients (7.4%) and, as the final option, the left basilic vein in 17 (6.7%).

There were 14 complications related to the procedure in our sample of patients, including two fractures of catheters with distal valves (0.8%), seven catheter obstructions (2.7%), six of polyurethane catheters without valves and one of a valved carbothane catheter, and there were five infections, all related to catheters without valves (1.9%).

Three different microorganisms were isolated: *Klebsiella pneumoniae* in three cases, *Candida Glabrata* in one case, and *Staphylococcus hominis* in one case. All infected catheters were in ICU patients.

## DISCUSSION

Robert B. Dawson delimited ideal zones for PICC insertion under ultrasonographic guidance (ZIM). Using musculoskeletal characteristics of the skin and vessels as landmarks, he divided the arm above the antecubital fold into three distinct zones, each 7 cm in size, separated by the colors red, green, and yellow, taking the medial epicondyle of the humerus as the initial anatomic landmark and the axillary line as the final landmark (Figure 5). In common with traffic lights, the colors of the zones indicate whether or not they should be used for puncture. According to Dawson, the ideal puncture zone, indicated with green, is approximately 12 cm from the medial epicondyle, where the basilic vein is most superficial in relation to the plane of the skin.^9^

**Figure 5:** Example measurement of the entire zone of approximately 21 cm divided into three 7 cm zones colored red, green and yellow.

In our study, we achieved a high rate of technical success with US-guided PICC insertion (96.1%). Our preference for ultrasound-guided puncture to achieve venous access was based on the lower risk of incorrect puncture offered by the ultrasonographic method when compared with puncture based exclusively on anatomic parameters.^10^^,^ 11 According to Hockley et al., the literature shows that US-guided insertion via the arm improves both catheter insertion success rates^12^^,^ 13 and the satisfaction of patients who undergo the procedure^14^ in addition to reducing complications, such as infections at the puncture site, thrombosis, and catheter migration.^15^

The most important complications of PICCs are: infection, fracture with distal venous migration, thrombophlebitis or DVT of upper extremities, Horner syndrome, and even chylothorax,^16^^-^ 18 the most commonly observed of which are infections, thrombophlebitis, and DVT.^5^^-^ 8

According to a study by Liem et al.,^19^ the rates of symptomatic upper limb superficial venous thrombosis associated with PICCs are 1.9% in the basilic vein, 7.2% in the cephalic vein, and 0% in the brachial vein. The greater incidence of superficial venous thrombosis in the cephalic vein is due to the anatomic characteristics of this vessel, such as smaller diameter in relation to the size of the catheter, lower number of tributaries, and more perpendicular insertion into the axillary vein (Figure 6).

**Figure 6:** Smaller diameter of the cephalic vein (white arrow) with relation to the size of the catheter and its perpendicular insertion into the axillary vein (black arrow).

For this reason, our team decided that the cephalic vein as the only available access option on either side would be considered an exclusion criterion, and chose to fit an SICVC in these cases.

A review of published retrospective and prospective studies revealed incidence rates of upper limb DVT associated with PICCs varying from 0.5 to 19.4%, with the higher incidence rates directly related to insertion of larger diameter PICCs and presence of malignant neoplasms. Just one cancer patient in our sample (who also had lower limb DVT) had an upper limb DVT, related to use of a nonvalved catheter and treated with removal of the catheter, with no need for subcutaneous or oral anticoagulation.

Compared with nonvalved PICCs, PICCs with integrated valve technologies significantly reduce the rates of later complications (occlusion or infection) and eliminate the need to use heparin and its potential subsequent complications (for example, heparin-induced thrombocytopenia).^20^

A retrospective study conducted by the Vanderbilt University involving placement of 12,505 devices and comparing rates of infection and occlusion in valved (4.2% and 1.4% respectively) and nonvalved PICCs (5.5% and 6.3% respectively), concluded that valved PICCs exhibited lower rates of infection and occlusion, a reduced need for maintenance, and lower costs, eliminating the obligatory heparin locking demanded for nonvalved PICCs.^21^ The results of our study, in which seven obstructions were described (six in nonvalved catheters and just one in a valved catheter) are in agreement with these data published in the literature.^20^^,^ 21

Peripherally inserted central catheters with proximal and distal valves were introduced to the market with the aim of reducing catheter occlusions by preventing blood backflow.^22^ A randomized prospective study conducted by Hoffer et al.^22^ showed that patency rates were better for catheters with proximal valves, with lower incidence rates of occlusive and infectious complications compared with catheters with distal valves.

The most common complication during insertion is malpositioning of catheters, which occurs when the catheter is not placed in the appropriate position within the vena cava.^23^ Difficulty advancing the catheter during insertion, inadequate blood draw, and difficulties removing the stylet/dilator sheath are indicative that the catheter may have been incorrectly placed and in such cases radiography or fluoroscopy is indispensable to identify whether the catheter has been incorrectly placed.^24^

In a study with 3,012 patients, conducted by Song and Li,^23^ technical success was achieved in 94.6% of PICC placements and 237 devices were identified as incorrectly placed outside of the vena cava, assessed by radiography after insertion of the catheter, with the most frequent location being the jugular vein, followed by the axillary and brachial veins (Figure 7). At our service, following a preestablished protocol, we did not detect any incorrectly positioned catheters. If fluoroscopy showed that the catheter was in a different position than the correct central position, it was immediately repositioned.

**Figure 7:** Path of catheter incorrectly positioned in the internal jugular vein.

When a catheter becomes obstructed, it is necessary to initiate the appropriate drug-based treatment with thrombolytics, attempting to reduce the degree of catheter obstruction. Baskin et al.^25^ conducted a study showing that thrombolytic agents successfully eliminate catheter occlusions in the majority of cases, and highlighted the role played by alteplase. Described as a safe and effective medication for deobstructing catheters, the disadvantages of this drug is its elevated cost, the fact that it is less effective than urokinase during the first 30 minutes of infusion, and that it takes more than 4 hours to achieve clearance of the catheter. Other thrombolytics require shorter periods of time in the lumen of the vessel to act.^25^ In our sample of patients, the obstructions observed in six nonvalved catheters and one proximally valved catheter were adequately treated using urokinase (Taurolock®), the thrombolytic of choice at our institution.

Maki et al.^26^ analyzed the risk of bloodstream infection with different types of device, finding that the bloodstream infection rates associated with PICCs were lower than those reported with traditional, non-tunneled, central venous devices.

Many hypotheses have been raised to attempt to explain why PICCs exhibit fewer infectious complications than other types of device, including lower bacterial density on the skin of the arm, the cooler temperatures of the limbs, and the relative simplicity of caring for the area, compared with the neck and groin.^6^ The following variables are related to bloodstream infections associated with PICCs: length of hospital stay, admission to an ICU, and number of catheter lumens.^27^ Sundriyal et al.^28^ analyzed placement of 246 PICCs in ICUs, observing that in 12.5% there were catheter infections with positive blood culture, in which the most frequently isolated agents were *Klebsiella pneumoniae* and *Staphylococcus sp.* These authors suggest that infected catheters should be removed in all patients with positive blood cultures, primarily if there is no improvement in fever within 48 h of appropriate administration of antibiotics.^28^ Fungemia or bacteremia caused by the *Bacillus* species *Corynebacterium jeikeium, Staphylococcus aureus, Pseudomonas aeruginosa* or *Stenotrophomonas maltophila* and nontuberculous mycobacteria will very often persist despite administration of appropriate antibiotics, in which case the catheter must be removed. Catheter removal should also be considered when blood cultures remain positive more than 48 h after treatment with antibiotics; if no other site of infection can be identified, or if bacteremia recurs after a course of antibiotics is completed.^29^

In our patient sample there were five cases of catheter infection leading to removal because of the principal agents identified: *Klebsiella pneumoniae, Candida glabrata* and *Staphylococcus hominis*. The results observed in our study are in line with the literature.

## CONCLUSIONS

Placement of peripherally inserted central venous catheters under ultrasound guidance and with fluoroscopy positioning offers a low incidence of complications and reduced infection rates and is safe and effective, particularly in cases with difficult vascular access, so these catheters are considered the first-choice devices for central vascular access. Their maintenance requires rigorous training of the nursing team, who should only be responsible for preserving, caring for, and protecting the catheter, with the objective of minimizing complications caused by incorrect handling. The placement procedure should be performed by a trained physician who is able to manage and resolve possible complications related to insertion and use of the catheter.
